# Clinical and genetic analysis of a case of late onset carbamoyl phosphate synthase I deficiency caused by *CPS1* mutation and literature review

**DOI:** 10.1186/s12920-023-01569-w

**Published:** 2023-06-26

**Authors:** Shangyu Wang, Jinglin Chen, Xiaoqi Zhu, Tingting Huang, Haifeng Xu, Guohuan Ying, Hao Qian, Wenxin Lin, Yiehen Tung, Kaleem Ullah Khan, Hu Guo, Guo Zheng, Haiying Lu, Gang Zhang

**Affiliations:** 1 Department of Neurology, Children’s Hospital of Nanjing Medical University, No.72 Guangzhou Road, Nanjing, Jiangsu China; 2 Nanjing Medical University, Nanjing, China

**Keywords:** Hyperammonemia, *CPS1* gene variant, Emerging mutations, Urea cycle disorder/carbamoyl phosphate synthase I deficiency

## Abstract

**Background:**

Carbamoyl phosphate synthetase I defect (CPS1D) is a rare disease with clinical case reports mainly in early neonates or adults, with few reports of first onset in late neonatal to childhood. We studied the clinical and genotypic characteristics of children with childhood onset CPS1D caused by two loci mutations (one of these is a rarely reported non-frame shift mutation) in the *CPS1*.

**Case presentation:**

We present a rare case of adolescent-onset CPS1D that had been misdiagnosed due to atypical clinical features, and further investigations revealed severe hyperammonemia (287µmol/L; reference range 11.2 ~ 48.2umol/L). MRI of the brain showed diffuse white matter lesions. Blood genetic metabolic screening showed elevated blood alanine (757.06umol/L; reference range 148.8 ~ 739.74umol/L) and decreased blood citrulline (4.26umol/L; reference range 5.45 ~ 36.77umol/L). Urine metabolic screening showed normal whey acids and uracil. Whole-exome sequencing revealed compound heterozygous mutations in the *CPS1*, a missense mutation (c.1145 C > T) and an unreported de novo non-frame shift mutation (c.4080_c.4091delAGGCATCCTGAT), respectively, which provided a clinical diagnosis.

**Conclusion:**

A comprehensive description of the clinical and genetic features of this patient, who has a rare age of onset and a relatively atypical clinical presentation, will facilitate the early diagnosis and management of this type of late onset CPS1D and reduce misdiagnosis, thus helping to reduce mortality and improve prognosis. It also provides a preliminary understanding of the relationship between genotype and phenotype, based on a summary of previous studies, which reminds us that it may help to explore the pathogenesis of the disease and contribute to genetic counselling and prenatal diagnosis.

## Background

**Fig. 1:**
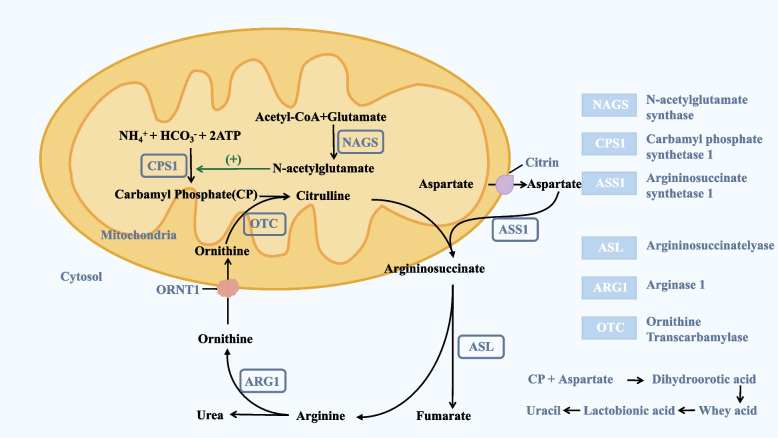
Diagram of urea cycle

Carbamoyl phosphate synthase I deficiency (CPS1D) is an autosomal recessive inherited metabolic disorder which is one of the rarer types of urea cycle disorder (UCD), with sudden onset, rapid deterioration, atypical symptoms, low morbidity, death before diagnosis and a poor prognosis for survivors. The incidence of this type of disease is approximately 1/50,000 ~ 1/300,000 and can be classified as neonatal onset or late onset depending on the age of onset. Previous clinical cases have reported that the neonatal type is more common, with severe clinical manifestations and extremely high mortality [1, 2], with fewer reports of late onset cases. More importantly, the clinical presentation of children with late onset disease is more unspecific and can be easily misdiagnosed. Furthermore, as the normal function of the CPS1 enzyme requires N-acetylglutamate as a metabolic activator, deficiency of *CPS1* and N-acetylglutamate synthase can exhibit the same biochemical alterations (Fig. 1), making genetic testing the gold standard for the diagnosis of CPS1D.

In this case, we have clinically, biochemically and molecularly characterized a rare child with late onset CPS1D and identified for the first time a new and rare non-frame shift mutation locus that may explain the late onset and good prognosis of this child. This result expands the mutational spectrum of the *CPS1*, facilitates early identification and genetic counselling of this patient population, and will alert us to explore potential mechanisms of disease pathogenesis.

## Case presentation

**Fig. 2:**
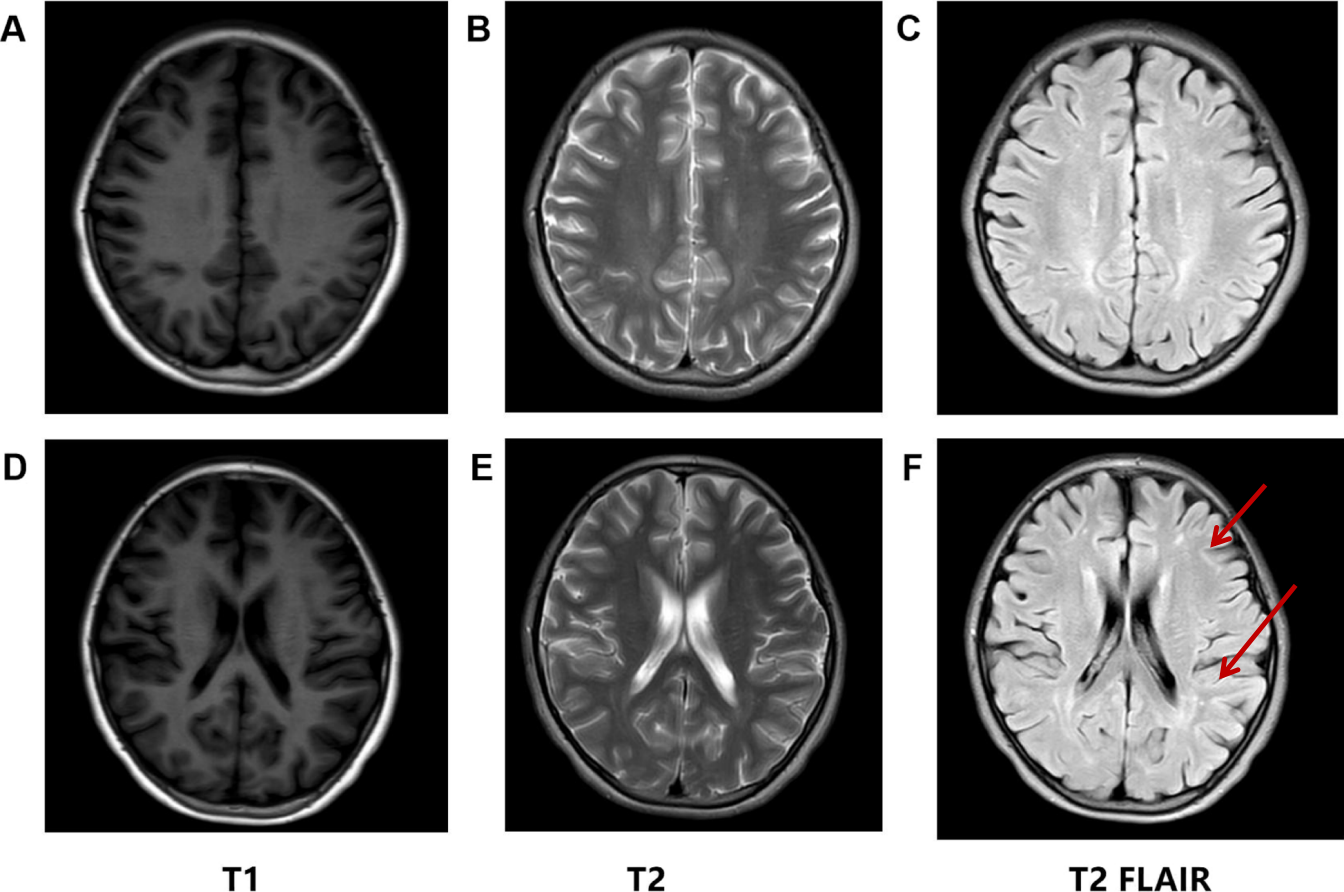
Brain MRI of the patient at the time of the first study. Brain MRI (3.0T) were performed at the Children’s Hospital of Nanjing Medical University. Axial T1 images (**A**, **D**), T2 images (**B**, **E**), and T2 fluid-attenuated inversion recovery images (**C**, **F**). **B**, **E** Small lamellar slightly long T2 signal shadow in the white matter of the frontoparietal brain on both sides, and high signal intensity on T2 FLAIR (**C**, **F**) images involving the white frontoparietal white matter on both sides (red arrows)

A 13-year-old and 6-month-old girl was brought to our attention because she was hospitalized twice in our hospital (Table 1). The first complaint was “fever and vomiting for 2 days with abnormal mental behavior for 1 day”. The fever peaked at 39.2℃. 1 day earlier, she suddenly babbled, had disorientation of persons and places, was markedly irritable, and alternated with delirium indifference. Cerebrospinal fluid routine, biochemical, pressure, virus, and culture were negative. Cranial MRI suggested multiple punctate abnormal signal shadows in the white matter of the frontoparietal brain on both sides, considering the possibility of intracranial infectious lesions, diagnosed as “viral encephalitis”. After 10 days of treatment with acyclovir antiviral, dexamethasone anti-inflammatory, supplemented with mannitol dehydration to lower cranial pressure, the above symptoms soon disappeared. However, a review of liver function showed that alanine aminotransferase (ALT436U/L; reference range 5 ~ 40U/L) and aspartate aminotransferase (AST96.7 U/L; reference range 5 ~ 40 U/L) indicators were elevated, suggesting liver function impairment, and liver ultrasound indicated liver enlargement, so acyclovir was discontinued and liver-protective treatment was given, while hepatitis virus was perfected and blood ammonia was sent. However, because the blood ammonia needed to be sent for out-of-hospital testing at that time, which was a cumbersome process, and because the parents believed that the child’s liver function and condition had improved significantly, they temporarily refused. After 1 week of hepatoprotective treatment, liver function and head MRI were normal, so the child was discharged on oral hepatoprotective medication. More than two years later, the child was readmitted with “vomiting for 1 week and headache with poor mental response for 3 days”, after having been treated for 1 week at an external hospital for a proposed diagnosis of “viral encephalitis” with poor results, and was then referred to our hospital. History taken: The patient was G2P1, with no abnormalities recorded during birth. Her parents were healthy and non-consanguineous Chinese, but her mother had an unexplained miscarriage at 28 weeks of gestation in G1. The patient showed appropriate growth and normal psychomotor milestones, but with academic failure. Physical examination: drowsiness, poor mental status, normal development, non-specific cardiopulmonary and abdominal examination, negative neurological examination. The blood ammonia concentration was 287umom/L and hyperammonemia was considered at the moment. The EEG suggested a slow background, but the cerebrospinal fluid examination was not abnormal. In combination with an abdominal CT suggesting a slightly hypodense liver and brain MRI findings: abnormal nodal signal in the white matter of the brain (Fig. 2), genetic metabolic disease was considered. Further refinement of the blood genetic metabolic screening showed elevated blood alanine (757.06umol/L; reference range 148.8 ~ 739.74µmol/L) and decreased blood citrulline (4.26umol/L; reference range 5.45 ~ 36.77umol/L). Urine organic acid gas mass spectrometry showed normal whey acids and urea, suggesting impaired urea cycling. Immediately after admission, the child was given a low protein diet, arginine to promote ammonia excretion (100 ~ 200 mg/(kg-d)), coenzyme Q10 (10 ~ 20 mg/(kg-d)) and levocarnitine (30 ~ 200 mg/(kg-d)) to regulate metabolism, lactulose to improve ammonia metabolism, vitamin B complex to promote nerve repair, and intravenous fluids to The child was given intravenous fluids to promote urinary ammonia excretion. After 1 week of treatment, the child’s headache and vomiting resolved significantly and she was discharged with instructions to follow a strict low-protein diet and to recheck her blood ammonia regularly. During hospitalization to clarify the type of urea cycle disorder, further refinement of whole exome sequencing after seeking parental consent identified compound heterozygous mutations in *CPS1* (NM_001875), a missense mutation (c.1145 C > T, p. Pro382Leu) and an unreported de novo non-frame shift mutation (c.4080_c.4091delAGGCATCCTGAT, p.Lys1360_I le1364delinsLys), respectively.

**Table 1:** Clinical characteristics and laboratory tests of the children admitted to our hospital on two occasions

**Onset time**	**2018.01.14**	**2020.05.21**
**Clinical symptoms**		
** Fever**	+	-
Vomiting	+	+
Headache	-	+
Irritable	+	-
Dispirited	+	+
Coma	-	-
Poor appetite and sleep	+	+
Urination and defecation	Normal	Normal
**Arterial blood gas analysis**	Lost information	
PH (reference, 7.25–7.45)	-	7.348
PO2 (reference, 50-80mmHg)	-	Lost information
PCO2 (reference, 40-60mmHg)	-	Lost information
HCO3- (reference, 19-30mmol/L)	-	22.5
BE (reference, –3 to + 3mmol/L)	-	-1.3
Lac (reference, 0.5 to 2.2mmol/L)	-	1.8
**Three routine (blood,urine, feces)**	Normal	Normal
**Blood biochemical tests**	Normal	Normal
**Blood ammonia (reference, 11.2 ~ 48.2µmol/L)**	Not Sent	287↑
**Blood mass spectrometry profile**	Not done	
Citrulline (reference, 5.45–36.77µmol/L)	-	4.26↓
Alanine (reference, 148.80-739.74µmol/L)	-	757.06↑
**Urinary organic acids**	Not done	
Urinary orotic acid (reference, 0-2mmol/L)	-	0.0
Urinary uracil (reference, 0-8mmol/L)	-	0.0
**Cerebrospinal fluid**	Normal	Normal
**Cephalometric MRI(Abnormal signals in the white matter of the brain)**	+	+
**Abdominal Imaging (Hepatomegaly)**	+	+
**Electroencephalogram (Background activity slowing down)**	-	+
**CPS1 sequencing**	Not done	
Allele 1 (from father)	-	c.1145 C > T
Allele 2 (from mother)	-	c.4080_c.4091delAGGCATCCTGAT

### Mutational analysis and pathogenicity prediction

**Fig. 3:**
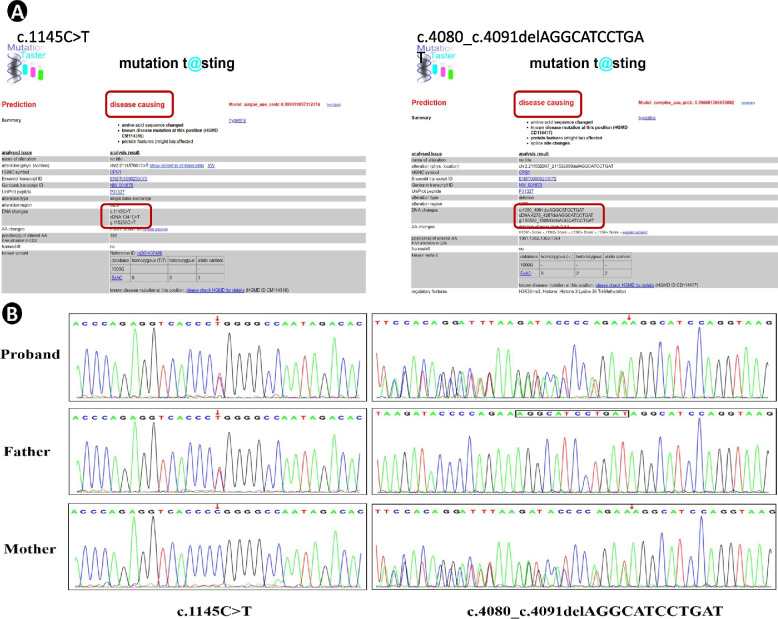
Pathogenicity was predicted using Mutation Taster (**A**) and two mutations in the patient and her family were confirmed by Sanger sequencing (**B**), i.e. c.4080_c.4091 delAGGCATCCTGAT heterozygous mutation carried by the mother and c.1145 C > T heterozygous mutation carried by the father

**Fig. 4:**
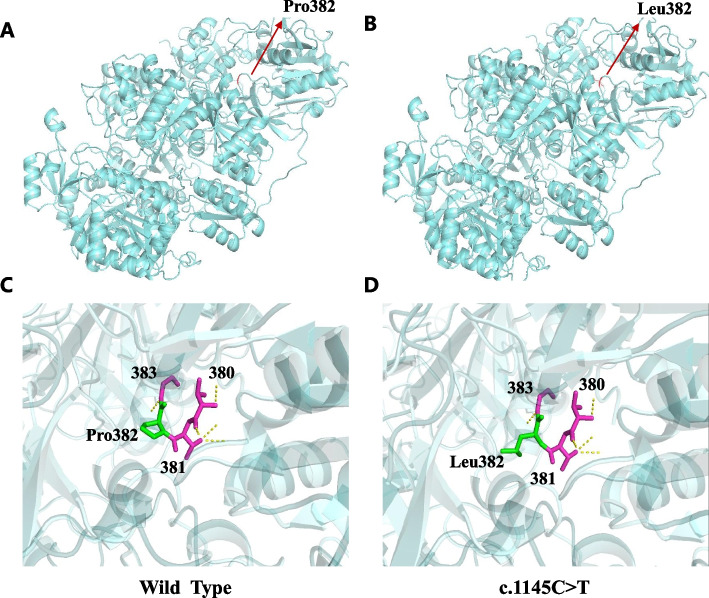
We constructed the above CPS1 models using the 6w2j.1.A human homology template in Swiss-Model software, respectively. The red marker in Figure **A** and the green marker in Figure **C** represent the 382 Pro position, the red marker in Figure **B** and the green marker in Figure **D** represent the 382 Leu position, the purple area in Figure **D** represents the amino acids and their functional residues near the missense mutation site, and the yellow areas in Figure **C** and Figure **D** represent the hydrogen bonding of amino acids to other sites. The mutation at the Pro position disrupts the primary and secondary structure of the original protein, and the amino acid loop at this position is ruptured, while the hydrogen bonding of the amino acid at this position to other amino acids is not significantly altered

**Fig. 5:**
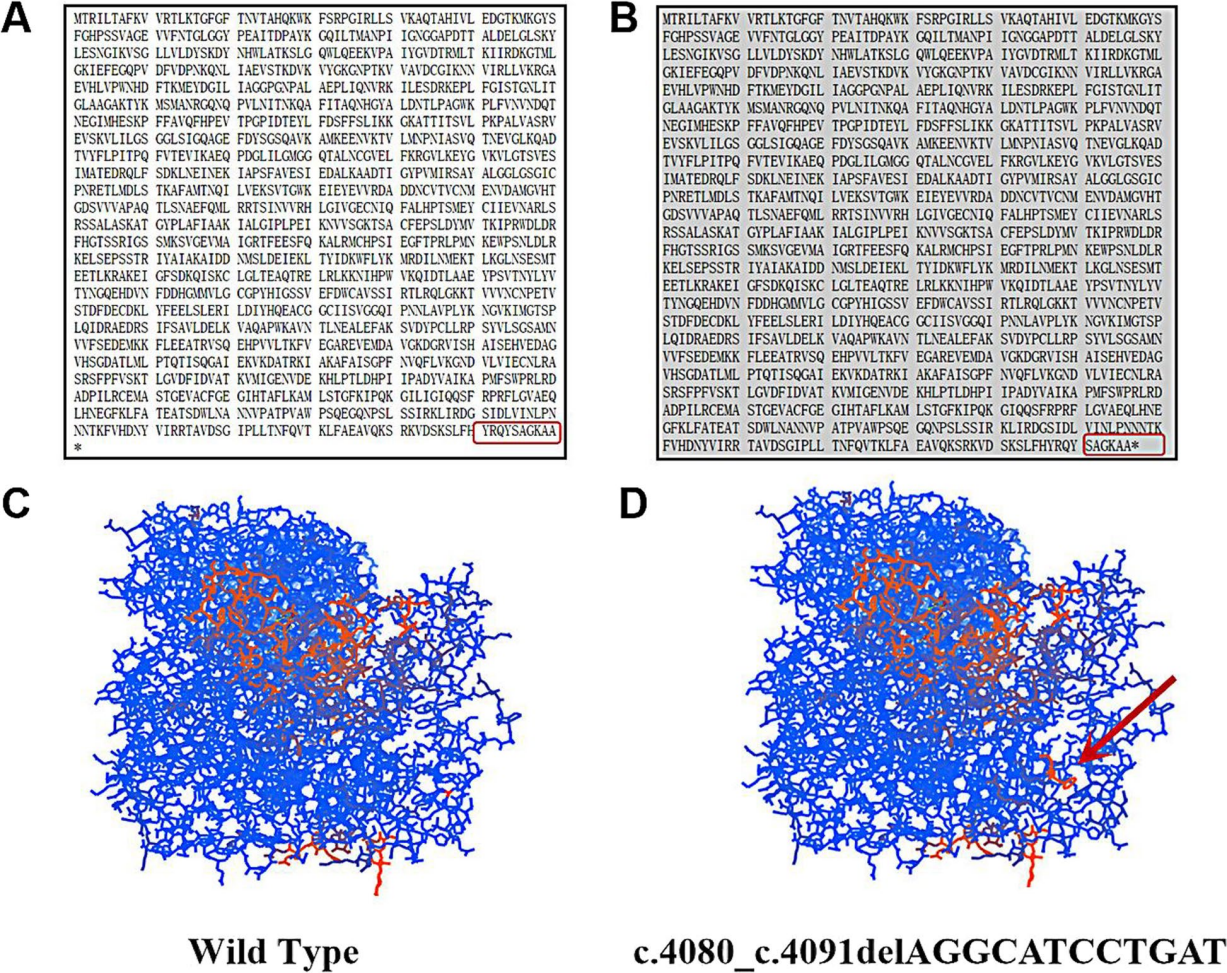
The c.4080_c.4091delAGGCATCCTGAT frameshift mutation did not result in a change in the reading frame, but only in an amino acid deletion, resulting in a change in protein length and structure. **A** is the wild type and **B** is the mutant. The protein prediction software SWISS-MODEL was used to predict the three-dimensional structure of the protein, with the red arrows pointing to the protein structure where the mutant differs from the wild type. **C** is the wild type and **D** is the mutant

*CPS1* is localized on chromosome 2q34 and contains 43 exons spanning over 120 kb and encoding 1500 amino acids [3]. We performed mutation screening using high-throughput sequencing technology, and all detected *CPS1* mutations were lineage verified by Sanger sequencing, aligned with the human genome (GRCh37 / hg19) reference sequence provided by the UCSC database, and compared with the currently known human *CPS1* sequence (National Center for Biotechnology Information, transcript number NM_001875). The database of mutant loci and single nucleotide polymorphisms (dbSNP) (http://www.ncbi.nlm.nih.gov/SNP/), the Human Gene Mutation Database (HGMD) (http://www.hgmd.cf.ac.uk/ac/index.php) and the Millennium Genome Database (http://browser.1000genomes.org/index.html) for comparative annotation. Finally, two compound heterozygous mutations were found in *CPS1* of this child, the missense mutation c.1145 C > T (p.Pro382Leu) (NM_001875) and the non-frame shift mutation c.4080_c.4091delAGGCATCCTGAT (p.Lys1360_Ile1364delinsLys) (NM_001875), where c.1145 C > T is a reported pathogenic mutation in CPS1D [4], while the non-shifted mutation c.4080_c.4091delAGGCATCCTGAT is found in OMIM, UCSC, HGMD, dbSNP, 1000 Genome, ExAC and gnomAD publications and public databases are new and not reported. The missense mutation c.1145 C > T results in an amino acid change from the non-polar amino acid proline (P) to the non-polar amino acid leucine (L), and the non-shift mutation c.4080_c.4091delAGGCATCCTGAT would result in an amino acid deletion at positions 1361–1364 (isoleucine-leucine-isoleucine-glycine), resulting in a protein length change. The pathogenicity of these two mutant loci was further analyzed using various prediction tools (SIFT, Polyphen2_HDIV, PROVEAN, MutationTaster, M-CAP, REVEL, GERP, phyloP20way, phastCons20way), Fig. 3A shows the pathogenicity using Mutation Taster for the pathogenicity prediction. Two variants were classified as “possibly pathogenic” according to the variant classification criteria of the American College of Medical Genetics and Genomics (ACMG 2015), and these variants were verified by Sanger sequencing in a family with a mother carrying the c. 4080_c.4091delAGGCATCCTGAT heterozygous mutation and a father with the c.1145 C > T heterozygous mutation (Fig. 3B), but without clinical manifestations, were consistent with an autosomal recessive disease pathogenesis pattern. Protein structure maps were generated using Swiss-pdb Viewer 4.10.The CPS1 protein structure of the missense mutation c.1145 C > T is shown in Fig. 4, and the CPS1 protein structure of the non-frame shift mutation c.4080_c.4091delAGGCATCCTGAT is shown in Fig. 5, with a theoretical 25% risk of disease in either fetus at the time of parental birth.

### Follow up

Although the child complied with a low protein diet for a short time after discharge and had regular blood ammonia checks, due to poor compliance, he was rushed to our PICU more than 8 months after his second discharge due to vomiting and coma after a large high-protein diet (hot pot), and in combination with the cause of this onset and the underlying disease of CPS1D, the emergency blood ammonia was 301 µmol/L (reference range 11.2 ~ 48.2umol/L). As the child was in a critical condition and in a coma, he was immediately given haemodialysis 7 times (1 time/day), fluid infusion, arginine and sodium benzoate to promote ammonia excretion, levocarnitine to promote metabolism, lactulose to reduce ammonia build-up in the intestine and B vitamins, as well as a ban on oral feeding and low amino acid intravenous nutrition. The child’s consciousness turned clear on the 7th day of treatment, the blood ammonia completely decreased to normal on the 43rd day, and he was discharged from the hospital on the 46th day. No hospital admissions for hyperammonemia at follow-up to date.

## Discussion and conclusions

**Fig. 6:**
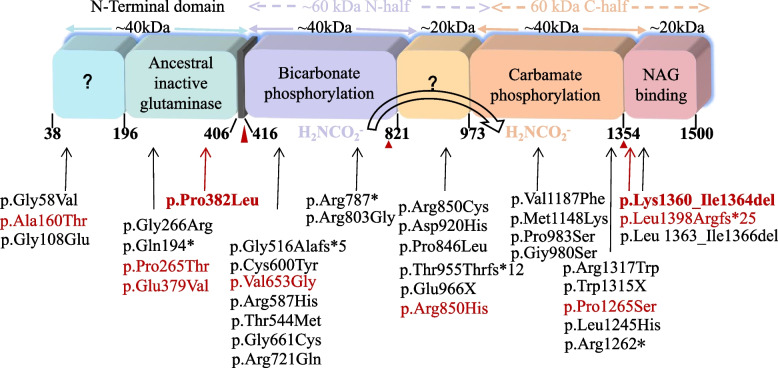
Structural basis of CPS1 function and defects. A bar chart consisting of the structural domains of the CPS1 peptide, color coded with the different structural domains, with arrows at the top of the bar chart indicating the position of all amino acid substitutions in Table 2, red font for late onset, red bold font for the mutation site in the child with this case, and black font for early onset, with the number of the first and last residue of each structural domain given at the bottom of the bar chart. The color codes of the different structural domains are also used in the other panels. The masses of the different structural domains, in kDa, are shown in black in the bar chart. The question marks indicate the so-called oligomeric structural domains of EcCPS

According to the The Human Gene Mutation Database (HGMD), more than 270 *CPS1* mutations have been reported internationally so far, but there are few cases in China [1, 5]. These mutations are unevenly distributed in the *CPS1*, which implies that certain regions are primarily responsible for enzyme folding and function. The CPS1 protein is divided into six domains (Fig. 6), of which two phosphorylation structural domains and the large subunit of the NAG-binding structural domain are important for enzyme activation, and mutation sites occurring at these sites are more likely to be pathogenic [2, 4].

**Table 2:** Summary of reported mutation loci in CPS1D cases in the last 5 years

Case	Variable loci	Structural Domain	Clinical Type	Ending	References
Case1	p.Gly58Val	N-Terminal	Early-onset	Survival (short-term)	Chen et al., 2018 [6]
	p.Gly266Arg				
Case2	p.Pro983Ser	Carbamate phosphorylation	Early-onset	Death	Bai et al., 2022 [7]
	p.Arg850Cys	20 kDa unknown functional structural			
Case3	p.Pro846Leu	20 kDa unknown functional structural	Early-onset	Death	Zhang et al., 2017 [1]
	p.Met1148Lys	Carbamate phosphorylation			
Case4	p.Cys600Tyr	Bicarbonate phosphorylation	Early-onset	Death	Zhang et al., 2017 [1]
	p.Leu 1363_Ile1366del	NAG binding			
Case5	p.Gln194*	N-Terminal	Early-onset	Survival (short-term)	Choi R et al., 2017 [8]
	p.Gly516Alafs*5	Bicarbonate phosphorylation			
Case6	p.Asp920His	20 kDa unknown functional structural	Early-onset	Unknown	M.Sc et al., 2018 [9]
Case7	p.Pro265Thr	Ancestral inactive glutaminase	Late-onset	Survival (short-term)	Lin et al., 2022 [10]
	p.Leu1398Argfs*25	NAG binding			
Case8	p.Arg787*	Bicarbonate phosphorylation	Early-onset	Survival (short-term)	Sugiyama Y et al., 2020 [11]
	p.Val1187Phe	Carbamate phosphorylation			
Case9	p.Thr544Met	Bicarbonate phosphorylation	Early-onset	Death	Yan et al., 2020 [12]
	p.Gly661Cys				
Case10	p.Glu966X	20 kDa unknown functional structural	Early-onset	Death	Yan et al., 2020 [12]
Case11	p.Ala160Thr	N-Terminal	Late-onset	Survival (long-term)	Fan et al., 2020 [13]
	p.Pro382Leu	Ancestral inactive glutaminase			
Case12	p.Thr955Thrfs*12	20 kDa unknown functional structural	Early-onset	Survival (short-term)	Fan et al., 2020 [13]
	p.Arg1317Trp	Carbamate phosphorylation			
Case13	p.Val653Gly	Bicarbonate phosphorylation	Late-onset	Survival (long-term)	Fan et al., 2020 [13]
	p.Pro382Leu	Ancestral inactive glutaminase			
Case14	p.Trp1315X	Carbamate phosphorylation	Early-onset	Survival (short-term)	Fan et al., 2020 [13]
	p.Arg587His	Bicarbonate phosphorylation			
Case15	p.Glu379Val	Ancestral inactive glutaminase	Late-onset	Survival (long-term)	Fan et al., 2020 [13]
	p.Pro1265Ser	Carbamate phosphorylation			
Case16	p.Arg850His	20 kDa unknown functional structural	Late-onset	Survival (long-term)	Ishikawa et al., 2022 [14]
Case17	p.Arg787X	Bicarbonate phosphorylation	Early-onset	Survival (long-term)	IMATAKA et al., 2021 [15]
	c.236 + 6T > C	-			
Case18	p.Arg721Gln	Bicarbonate phosphorylation	Early-onset	Survival (long-term)	Zhou et al., 2020 [16]
	p.Giy980Ser	Carbamate phosphorylation			
Case19	p.Leu1245His	Carbamate phosphorylation	Early-onset	Survival (long-term)	Zhou et al., 2020 [16]
	p.Arg1262∗				
Case20	p.Arg803Gly	Bicarbonate phosphorylation	Early-onset	Survival (short-term)	Yang et al., 2017 [5]
	p.Gly108Glu	N-Terminal			

The clinical presentation and biochemical response of this patient are consistent with the mutational findings, suggesting that the current understanding of the CPS1 protein structure can be used to explain the genetic mutation in this child. Genetic analysis of this patient revealed that c.1145 C > T (p.P382L) is located in the N-terminal subunit of the enzyme, and we summarized 20 case reports of CPS1D published in the open literature base over the past 5 years (Table 2), of which 5 were late onset cases (4 double allele variants and 1 single allele variant) [10, 13, 14], and all children with late double allele variants had a mutation in the N-terminal subunit. These cases had a good prognosis with onset at puberty, which is basically consistent with the “atypical” late onset features, suggesting that probably mutations in this region have no significant effect on enzyme function. In addition, analysis of the etiology from the site of the missense mutation may be related to the insignificant location of the mutation in the locus. Homology modeling by Swiss-Model showed that the mutation at position 382 Pro disrupted the primary and secondary structure of the original protein, resulting in a break in the amino acid loop at this position, but no significant change in the hydrogen bonding force between the amino acid at this position and other amino acids, which we speculate may also account for the late onset, good outcome and low neurological damage in our child. Similarly Swiss-Model shows that c.4080_c.4091delAGGCATCCTGAT (p.Lys1360_Ile1364delinsLys) is located in the NAG-binding structural domain of the CPS1 enzyme (Fig. 5), the deletion of four amino acids occurs at a highly evolutionarily conserved position with a lysine inserted, and a small deletion likely disrupts the conformation of the structural domain at the NAG binding site, thereby disrupting NAG binding to CPS1 and further inhibiting enzyme activation. To our knowledge, there are only four previous reports of small deletion mutations in *CPS1*. We conclude that although previous studies have shown that mutant sites are more likely to be pathogenic when located in the structural domain encoding the NAG binding site, not all mutation types result in severe clinical manifestations, especially when there is already a mutation located in the N-terminal structural domain. We hypothesize that in this case, although the NAG structural domain remains an entry site for ammonia substrates, it has little effect on the Km of ammonia in CPS1, while it cannot be excluded that these two mutant sites act as a mutual check and balance, however, further functional studies are still needed to elucidate the molecular pathogenesis of the new mutation.

In addition, using 20 case reports of CPS1D that could be found in publicly available databases over the past 5 years (Table 2), we initially explored the intrinsic genotype-phenotype relationship of CPS1D and identified all variant loci on the structural domain of the CPS1 enzyme (Fig. 6). The majority of the 20 patients had a double allele variant and a few cases (6, 10, 16;2 neonatal onset and 1 late) had a single allele variant, and all single allele variants occurred in the same 20 kDa unknown functional structure and had a variable prognosis [9, 12, 14]. This requires further studies to determine whether CPS1 activity in such patients could be influenced by certain acquired factors. In any case, this may have some clinical implications for the diagnosis and prognosis of this patient.

In conclusion, CPS1D is usually sporadic and difficult to diagnose due to the multi-organ nature of its clinical manifestations, mostly in the early fatal neonatal period [17], but atypical late onset cases should not be ignored. Therefore, in clinical practice, children with recurrent gastrointestinal symptoms, impaired consciousness, abnormal psychiatric behaviour, vegetarian habits and unexplained neuropsychological developmental delays should be alerted to such disorders and undergo early blood ammonia screening to minimize misdiagnosis. If hyperammonaemia is detected, early blood and urine amino acid analysis and genetic testing should be performed, thus helping to reduce mortality and improve prognosis. We first identified two mutations in *CPS1* that may counterbalance each other in a Chinese patient with a compound heterozygous mutation and comprehensively characterized their clinical features, which may facilitate our exploration of the underlying mechanisms of disease pathogenesis and thus further clarify the genotype and phenotype associated with the disease. This study also illustrates the impact of genetic counseling on families and contributes to the prevention of birth defects.

## Data Availability

The datasets used or analysed during the current study are available from the corresponding author on reasonable request.

## Abbreviations

CPS1D: Carbamoyl phosphate synthetase I defect
UCD: Urea cycle disorder
CP: Carbamoyl phosphate
NAG: N-acetylglutamate
HGMD: Human Gene Mutation Database
